# Interleukin-23 in lung and airway diseases: from pathogenesis to precision-guided therapeutic targeting

**DOI:** 10.3389/fphar.2026.1784434

**Published:** 2026-03-18

**Authors:** Barsha Baisakhi Nayak, Rishi Rajesh, Julia Teppan, Theresa Gogg, Eva Böhm

**Affiliations:** 1 Otto Loewi Research Center for Vascular Biology, Immunology and Inflammation, Division of Pharmacology, Medical University of Graz, Graz, Austria; 2 Lung Research Cluster, Medical University of Graz, Graz, Austria

**Keywords:** asthma, chronic obstructive pulmonary disease, corticosteroid resistance, COVID-19, idiopathic pulmonary fibrosis, interleukin-23, respiratory inflammation, targeted immunotherapy

## Abstract

Interleukin-23 (IL-23) is a pleiotropic cytokine belonging to the IL-12 family and is predominantly produced by antigen-presenting cells. It plays a central role in shaping adaptive immunity by promoting the polarization, expansion, and maintenance of T helper 17 (Th17) cells, thereby driving the production of downstream effector cytokines such as IL-17A and IL-22. Under physiological conditions, IL-23 contributes to pulmonary immune homeostasis and host defense against bacterial and fungal pathogens. However, sustained or dysregulated IL-23 signaling promotes chronic inflammation and tissue damage. Beyond autoimmune diseases, where IL-23 is a well-established key mediator linked to disease severity and a validated therapeutic target, it has also emerged as a critical mediator in chronic lung diseases. Enhanced IL-23 signaling has been associated with increased disease severity, corticosteroid resistance, airway remodeling, and progressive tissue fibrosis, highlighting its contribution to both inflammatory and structural components of lung pathology. These findings suggest that IL-23 is not merely a bystander but an active driver of pathogenic processes in the respiratory system. In this review, we synthesize recent advances in understanding the role of IL-23 in chronic obstructive pulmonary disease, asthma, idiopathic pulmonary fibrosis, and coronavirus disease 2019. We further discuss the therapeutic potential of targeting IL-23 as a precision-guided strategy to modulate respiratory inflammation and remodeling, with particular emphasis on corticosteroid resistance, fibrotic endotypes, safety and pharmacologic tradeoffs, and the emerging role of IL-23-related biomarkers and molecular endotyping for precision-guided patient stratification and targeted intervention.

## 1 Introduction

Lung inflammation arises primarily from exposure to allergens, pathogens, and environmental pollutants. It can be broadly categorized as acute, as seen in pneumonia and acute respiratory distress syndrome (ARDS), or chronic, as observed in asthma and chronic obstructive pulmonary disease (COPD) (Moldoveanu et al., 2009). The airway epithelium functions as the first line of defense against inhaled insults by trapping and neutralizing harmful agents through the secretion of mucins, defensins, lysozyme, lactoferrin, and nitric oxide (Adler et al., 1994). During inflammatory responses, epithelial cells further release proinflammatory cytokines and mediators, including tumor necrosis factor (TNF)-α, interleukin (IL)-1β, and reactive oxygen species, which recruit and activate immune cells within the pulmonary microenvironment. The ensuing cascade leads to excessive mucus secretion, airway hyperresponsiveness, and tissue injury (Adler et al., 1994). Persistent or unresolved inflammation promotes fibrotic remodeling, characterized by excessive extracellular matrix deposition, tissue scarring, and progressive loss of lung function (Wilson and Wynn, 2009). Among these mediators, IL-23, a heterodimeric cytokine of the IL-12 family produced mainly by antigen-presenting cells and epithelial cells, has emerged as a key upstream regulator of chronic inflammatory responses by sustaining T helper 17 (Th17) cell activity and downstream effector pathways, positioning it as a potential driver of persistent airway inflammation, corticosteroid resistance, and tissue remodeling.

Major respiratory disorders such as COPD, asthma, idiopathic pulmonary fibrosis (IPF), and COVID-19 collectively account for a significant global health burden. COPD, the third leading cause of death worldwide, is marked by irreversible airflow limitation and chronic neutrophilic inflammation (Wang Z. et al., 2025). Asthma affects over 300 million individuals globally and, while often manageable, severe or corticosteroid-resistant cases are prone to recurrent exacerbations (GBD, 2019 Diseases and Injuries Collaborators, 2020; Yang et al., 2024). IPF, though relatively rare, is a devastating interstitial lung disease with a median survival of only 3–5 years following diagnosis (Nalysnyk et al., 2012; Martinez et al., 2017; Lederer and Martinez, 2018). The COVID-19 pandemic, caused by SARS-CoV-2, has further underscored the global impact of respiratory inflammation, leading to millions of deaths worldwide (Parasher, 2020). Across these clinically distinct disorders, overlapping immunopathological features, including neutrophilic inflammation, epithelial barrier disruption, maladaptive repair, and treatment-refractory disease courses, suggest shared upstream inflammatory drivers that are not adequately addressed by current therapies. Notably, IL-23 occupies an upstream position within these networks by stabilizing pathogenic Th17 responses and amplifying IL-17- and IL-22–mediated effects on epithelial cells, fibroblasts, and innate immune populations, thereby linking inflammatory endotypes with fibrotic remodeling and impaired therapeutic responsiveness.

Collectively, these conditions highlight the urgent need to deepen our understanding of inflammatory lung disease mechanisms. In this review, we discuss the immunopathological pathways underlying these disorders, with a particular emphasis on the role of IL-23 signaling and its emerging therapeutic potential. Although IL-23 is best known for its pathogenic role in autoimmune diseases, accumulating evidence suggests that IL-23–driven pathways may also contribute to pulmonary inflammation and fibrosis, making this axis a promising target for precision-guided intervention in chronic lung disease. We frame IL-23 targeting within a precision medicine paradigm, emphasizing endotype-specific, biomarker-informed patient stratification rather than one-size-fits-all application. This approach aims to identify biologically defined patient subgroups most likely to benefit from IL-23 pathway modulation while minimizing unnecessary immunosuppression and infection risk.

This review advances the field by (i) integrating evidence across asthma, COPD, IPF, and COVID-19 to position IL-23 as a shared upstream driver of neutrophilic and fibrotic lung endotypes; (ii) critically evaluating preclinical and emerging clinical data on IL-23/Th17-targeted therapies in pulmonary disease; and (iii) proposing a precision-guided framework for patient stratification based on inflammatory endotypes and biomarker profiles. By unifying mechanistic insights with translational and therapeutic perspectives, this review aims to clarify when and in whom IL-23 pathway modulation is most likely to be beneficial.

### 1.1 Cytokines, immune effector cells, and therapeutic limitations in inflammatory lung diseases

#### 1.1.1 Cytokines in inflammatory diseases

Cytokines play a crucial role in regulating inflammation and are therefore dysregulated in inflammatory lung diseases. Proinflammatory cytokines such as TNF-α, IL-1β, and IL-6 promote inflammation by activating immune cells and increasing vascular permeability, whereas anti-inflammatory cytokines, such as IL-10, resolve inflammation and support tissue repair. An imbalance between pro- and anti-inflammatory cytokines contributes to disease progression (Moldoveanu et al., 2009). In allergic asthma, cytokines such as IL-4, IL-5, and IL-13 enhance immunoglobulin (Ig) E production and eosinophil activation, leading to persistent airway inflammation and remodelling (Holgate, 2008), while TNF-α, interferon (IFN)-γ, IL-1β, IL-6, and GM-CSF are associated with COPD (Sarir et al., 2008). Similarly, TNF-α, IFN-γ, IL-1β, IL-6, IL-2, IP-10, and CXCL8/IL-8 are among the hallmark cytokines most consistently elevated in coronavirus disease 2019 (COVID-19) ‘cytokine storm’ (Ghaffarpour et al., 2025; Zanza et al., 2022), while in idiopathic pulmonary fibrosis (IPF), profibrotic transforming growth factor (TGF)-β1 and proinflammatory TNF-α, IL-6, and IL-1β contribute to excessive scarring of lung tissue (Wynn, 2008), characterized by elevated extracellular matrix deposition that impairs lung function and worsens clinical outcomes (Noble and Homer, 2004; Richeldi et al., 2017).

#### 1.1.2 Immune cells in lung diseases

Immune cells play a key role in the development and progression of lung diseases, contributing to both inflammation and fibrosis. Dendritic cells (DCs) are involved in antigen processing and presentation to T cells, thereby initiating the immune response in diseases such as asthma and chronic bronchitis (Lipscomb et al., 1995). In asthma, CD4^+^ T cells promote allergic inflammation, whereas CD8^+^ T cells are more common in COPD (Barnes, 2008). Macrophages, depending on their phenotype, play a dual role in promoting inflammation and fibrosis and are abundantly present in the lungs (Wynn and Vannella, 2016). In COPD, macrophages contribute to persistent inflammation and tissue damage (Hogg and Timens, 2009), while in IPF, they promote fibroblast activation and collagen deposition (Byrne et al., 2015). Neutrophils are essential in acute inflammation, as they quickly respond to infections and cause tissue damage by producing reactive oxygen species (Kolaczkowska and Kubes, 2013), but are also involved in chronic lung diseases such as non-allergic asthma, COPD, and IPF. Eosinophils are not only crucial players in allergic conditions and asthma, but also are involved in releasing cytokines, growth factors, and enzymes (like IL-4, IL-13, TGF-β1, and EPO) that contribute to fibrosis (Rothenberg and Hogan, 2006). Upon activation, tissue-resident mast cells secrete histamines, proteases, and leukotrienes, contributing to both acute hypersensitivity and chronic inflammation (Prussin and Metcalfe, 2006). Monocytes, macrophages (Merad and Martin, 2020) and neutrophils (Xu et al., 2022) are also the dominant proinflammatory cells in COVID-19, driving cytokine storm and lung damage.

#### 1.1.3 Current treatment recommendations and limitations

Across diseases, inhaled corticosteroids and bronchodilators form the backbone of asthma and COPD management, while selected biologics targeting type 2 inflammation improve outcomes in defined asthma endotypes. However, a substantial proportion of patients exhibit incomplete responses, corticosteroid resistance, or treatment-limiting adverse effects, and biologics remain costly and unevenly accessible (Castro et al., 2015; Chung et al., 2014). In COPD, available therapies reduce symptoms and exacerbations but do not halt progressive declines in lung function, and long-term corticosteroid use does not benefit all patients and increases the risk of infections (Rabe et al., 2005; Vogelmeier et al., 2017). In IPF, approved antifibrotic agents (pirfenidone and nintedanib) slow disease progression but are not curative and are frequently limited by adverse effects and high costs (King et al., 2014; Richeldi et al., 2014). Similarly, immunomodulatory therapies used in severe COVID-19 can attenuate hyperinflammation but do not address the underlying drivers of lung injury and repair, and may carry risks related to immune suppression (National Institute for Health and Care Excellence Great Britain, 2021).

Collectively, these limitations highlight a central unmet need: current therapies largely manage symptoms and downstream inflammatory consequences but fail to adequately target upstream immunopathological drivers of chronic lung inflammation and tissue remodeling. This therapeutic gap is further compounded by disease heterogeneity, treatment resistance, and the lack of robust biomarkers to guide patient stratification and therapeutic selection.

#### 1.1.4 Historical perspective on IL-23

In the late 1990s, it was found that using IL-12p40 neutralizing antibodies or disrupting the IL-12p40 gene to block IL-12 signaling provides protection in models of autoimmune diseases (McIntyre et al., 1996; Constantinescu et al., 1950; Segal et al., 1998). However, targeting the other IL-12 subunit, IL-12p35 worsened disease outcomes (Cua et al., 2003). This was clarified through a computational sequence screening, which revealed that the IL-12p40 subunit can dimerise not only with IL-12p35, but also with another subunit, p19. The resulting heterodimer composed of IL-12p40 and p19 was termed IL-23. Thus, a novel member of the IL-12 cytokine family, now known as IL-23, was discovered by Oppmann et al., in 2000 (Oppmann et al., 2000).

Following its discovery, IL-23 was rapidly recognized as a key driver of pathogenic Th17 immunity and chronic inflammation in autoimmune diseases, which catalyzed therapeutic development targeting the IL-12/IL-23 axis. The first major clinical milestone was the FDA approval of the IL-12/23p40–targeting monoclonal antibody ustekinumab in 2009 for moderate-to-severe plaque psoriasis (Leonardi et al., 2008), followed by the approval of IL-23p19–specific inhibitors such as guselkumab in 2017 (Megna et al., 2018), establishing selective IL-23 blockade as an effective therapeutic strategy in immune-mediated inflammatory diseases.

In parallel with these clinical advances, early experimental studies began to implicate IL-23/Th17 signaling in respiratory inflammation, including murine models of allergic asthma (Li et al., 2011) and neutrophilic airway disease (Wang et al., 2012), followed by translational observations linking elevated IL-23 and IL-17 levels to disease severity in COPD (Rong et al., 2020) and IPF (Weng et al., 2019; Wilson et al., 2010). These findings expanded the relevance of IL-23 biology beyond autoimmunity to chronic inflammatory and fibrotic lung diseases and provided the conceptual basis for exploring IL-23–targeted strategies in respiratory medicine.

#### 1.1.5 Cellular sources and regulation of IL-23 synthesis

IL-23 is a cytokine that plays an important role in the immune system. It is classically secreted by antigen-presenting cells (APCs) such as dendritic cells, macrophages, and monocytes (Wilson et al., 2007) in response to pathogen-associated molecular patterns and other immune signals, and contributes to the T helper (Th) 1 and Th17 immune response (Oppmann et al., 2000; Wilson et al., 2007; Teng et al., 2015). This process is essential for initiating and maintaining immune responses, particularly in inflammation and autoimmunity (Cua et al., 2003).

Recent research has expanded the understanding of IL-23 sources during chronic inflammatory diseases and infections. Notably, airway epithelial cells have been shown to produce IL-23, particularly in respiratory diseases such as COPD and severe asthma (Lee et al., 2017). Upon chronic exposure to proinflammatory mediators or acute viral infections, these epithelial cells release IL-23, promoting the activation and expansion of Th17 cells (Lee et al., 2017). Similarly, keratinocytes in skin diseases (Yoon et al., 2016) and even neutrophils, under certain inflammatory conditions, have been observed as sources of IL-23 (Kvedaraite et al., 2016; Wang and Shi, 2023). This broadens the role of IL-23 beyond its classical sources, highlighting its involvement in pathological immune responses across different tissues. The production of IL-23 by non-APC sources like epithelial cells underscores its role in the development of chronic inflammatory diseases, making it a relevant target for therapies aimed at regulating inflammatory responses in these conditions.

#### 1.1.6 Structure, receptors, and signaling pathways of IL-23

The IL-23 signaling cascade is a tightly regulated pathway that plays a central role in the differentiation and function of Th17 cells, which are key players in autoimmunity and inflammation. The cascade begins with the binding of IL-23, a heterodimeric cytokine composed of two subunits: p19, which is unique to IL-23, and p40, which is shared with IL-12 (Oppmann et al., 2000), to the IL-23 receptor complex, a heterodimer consisting of IL-23R and IL-12Rβ1 (Oppmann et al., 2000; Parham et al., 1950). This receptor is expressed on various immune cells, including memory T cells, natural killer (NK) cells, dendritic cells, Th17 cells, and innate lymphoid cells (ILCs), particularly ILC3. Upon release from antigen-presenting cells such as dendritic cells and macrophages, IL-23 binds to its receptor complex, initiating the intracellular Janus kinase/signal transducer and activator of transcription (JAK/STAT) signaling pathway (Hunter, 2005). The receptor-ligand interaction leads to autophosphorylation and activation of JAK2 and TYK2, which then phosphorylate specific tyrosine residues on the cytoplasmic tails of the IL-23 receptor subunits (Hunter, 2005). These phosphorylated residues act as docking sites for STAT proteins, which are essential transcription factors in cytokine signaling (Hunter, 2005).

Among the STAT proteins, STAT3 is recruited to the phosphorylated receptor complex (Gaffen et al., 2014). Upon phosphorylation, STAT3 dimerizes and translocates into the nucleus. There, it binds to specific DNA sequences in the promoter regions of target genes, initiating the transcription of genes involved in inflammatory responses, cell survival, proliferation, and differentiation (Gaffen et al., 2014).

The IL-23 signaling pathway is modulated by interactions with other cytokine pathways, maintaining the balance between protective immunity and harmful autoimmunity. For instance, IL-6 and TGF-β are involved in the initial differentiation of Th17 cells, while IL-23 is essential for their expansion, promotion, and pathogenicity (Dong, 2008). Additionally, the pathway is subject to regulatory feedback mechanisms, such as the induction of Suppressor of Cytokine Signaling (SOCS) proteins. For example, SOCS3 binds to JAK2 or the IL-23 receptor complex, thereby blocking STAT3 activation and inhibiting further signaling to prevent excessive inflammation (Yoshimura et al., 2007; Chen et al., 2006). These regulatory mechanisms ensure a controlled immune response and limit potential tissue damage.

### 1.2 IL-23 target cells

#### 1.2.1 Th17 cell differentiation and promotion of inflammation

**FIGURE 1:**
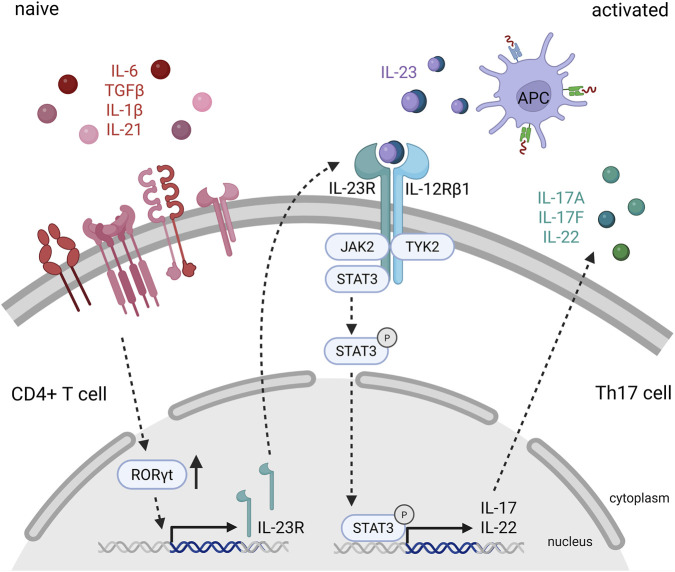
Mechanistic signaling of IL-23 in Th17 cell activation and cytokine production. Naïve CD4^+^ T cells differentiate into Th17 cells under the influence of IL-6, IL-1β, TGF-β, and IL-21. These cytokines collectively induce the expression of the lineage-defining transcription factor RORγt and upregulate IL-23 receptor (IL-23R) expression. Activated antigen-presenting cells (APCs) secrete IL-23, which binds to the IL-23R/IL-12Rβ1 receptor complex on Th17 cells, leading to the activation of the JAK2/TYK2 signaling cascade and subsequent phosphorylation of STAT3. Activated STAT3 enhances the transcription of key Th17 effector cytokines, including IL-17A, IL-17F, and IL-22. These cytokines promote airway inflammation by stimulating epithelial cells and recruiting neutrophils to the respiratory tract, thereby amplifying the inflammatory response.

While Th17 cells contribute to protection and tissue homeostasis under physiological conditions (Agalioti et al., 2023), their activation in the presence of IL-23 leads to sustained inflammation and ongoing tissue damage. In combination with other cytokines such as IL-6, TGF-β, IL-1β, and IL-21, IL-23 promotes the differentiation of naive CD4^+^ T cells into Th17 cells (Figure 1). T cell receptor engagement in response to external stimuli, along with cytokine signals from TGF-β, IL-1, and IL-6, activates the master transcription regulator Retinoic acid receptor-related orphan receptor gamma t (RORγt) to initiate Th17 polarization and drives Th17 family cytokine and IL-23R expression (Teng et al., 2015; Ivanov et al., 2006). This is followed by IL-23 interacting with IL-23R, which activates the transcription factor STAT3 and further promotes the transcription of IL-23R and RORγt. Thus, by creating a positive feedback loop, IL-23 induces a more pathogenic phenotype in Th17 cells by supporting Th17 polarization and survival (Teng et al., 2015), as well as the expression of proinflammatory cytokines, while suppressing anti-inflammatory cytokines such as IL-10 (McGeachy et al., 2007).

Upon activation by IL-23, Th17 cells produce a range of cytokines including IL-17A, IL-17F, IL-22, GM-CSF and TNF-α (Gaffen et al., 2014). These cytokines mediate neutrophilic inflammation by promoting the recruitment of neutrophils to sites of infection and tissue damage (Gaffen et al., 2014; Lubberts, 2015). IL-17A and IL-17F are potent inducers of chemokines such as IL-8/CXCL8, which attract neutrophils and amplify the inflammatory response (Lubberts, 2015; Al-Ramli et al., 2009). While the neutrophilic response is important for host defense against infections, uncontrolled IL-23-driven Th17 polarization can lead to chronic inflammation in autoimmune diseases such as psoriasis, rheumatoid arthritis, inflammatory bowel disease, and multiple sclerosis. In these conditions, persistent recruitment and activation of neutrophils by Th17 cytokines promote tissue damage and disease progression (Wang and Shi, 2023; Moschen et al., 2019; Abdo and Tye, 2020). IL-23-dependent Th17 polarization also plays a role in chronic lung inflammation, including COPD and severe asthma. In the lungs, Th17-derived cytokines cause sustained neutrophil recruitment and activation, resulting in chronic neutrophilic activation, persistent airway inflammation, remodeling, and progressive impairment of lung function (Al-Ramli et al., 2009; Alcorn et al., 2010).

#### 1.2.2 IL-23 target cells beyond CD4^+^ and Th17 cells

Although Th17 cells are the prototypical responders to IL-23, a variety of innate and innate-like lymphocytes also express IL-23R and contribute to IL-23–driven inflammation (Table 1). These cell populations can respond rapidly in an antigen-independent manner, thereby amplifying local immune responses in the lung.

**TABLE 1:** IL-23 target cells besides CD4^+^ and Th17 cells.

Cell type	Key cytokines	Main functions	Associated lung diseases	Key mechanisms	References
ILC3	IL-17A, IL-17F, IL-22	Rapid mucosal inflammation; epithelial chemokine and antimicrobial peptide induction; neutrophil recruitment	Neutrophilic asthma; COPD; viral/bacterial infections; fibrotic remodeling	Antigen-independent activation; contributor to corticosteroid-resistant airway inflammation	Hunter (2005), Gaffen et al., 2014; Dong (2008), Yoshimura et al. (2007), Chen et al. (2006), Agalioti et al. (2023), Ivanov et al. (2006)
γδ T cells	IL-17A, IL-17F	Early neutrophilic responses during infection; maintenance of chronic neutrophilic inflammation; tissue repair	Viral infections; chronic airway inflammation; IPF	Context-dependent activation; pathogenic or protective roles	Hong et al. (2025), Pociask et al. (2011), Braun et al. (2008), Lo et al. (1950)
iNKT cells	IL-17, IL-22	Bridge innate and adaptive immunity; promote fibrosis via neutrophil recruitment, inflammasome activation, macrophage and fibroblast activation	IPF	Interact with type 1, 2, and 3 immune pathways; therapeutic potential via iNKT inhibition	Michel et al. (2007), Kumar et al. (2023), Kim et al. (2008), Syn et al. (2012), Crosby and Kronenberg (2018)

Innate lymphoid cells type 3 (ILC3) are important early sources of IL-17A, IL-17F, and IL-22 in response to IL-23 stimulation (Hunter, 2005; Gaffen et al., 2014). Unlike Th17 cells, ILC3s lack antigen-specific receptors and are activated directly by cytokines derived from myeloid cells, allowing them to mediate rapid inflammatory responses at mucosal surfaces. In the lung, IL-23–driven ILC3 activation has been implicated in neutrophilic asthma (Klose and Artis, 2016; Robinette et al., 2015; Kim et al., 2014), COPD (De Grove et al., 2016), viral and bacterial infections (Ardain et al., 2019; Hoffmann et al., 2021), and fibrotic remodeling through the production of IL-22 and IL-17 family cytokines (Ardain et al., 2019). These cytokines stimulate epithelial cells to produce chemokines and antimicrobial peptides, promoting neutrophil recruitment and epithelial activation even in the absence of adaptive immune activation. ILC3 responses may be particularly relevant in corticosteroid-resistant airway inflammation, where innate immune pathways dominate (Berkinbayeva et al., 2024).

γδ T cells represent another major IL-23–responsive population that plays a dual role in host defense and immunopathology. Upon stimulation with IL-23, lung-resident γδ T cells rapidly produce IL-17A and IL-17F, contributing to early neutrophilic responses during viral infections. However, their role in IL-23–driven chronic lung inflammation remains incompletely understood. On one hand, persistent IL-23–mediated activation of γδ T cells may promote chronic neutrophilic inflammation and tissue injury in the airways (Hong et al., 2025). On the other hand, several studies have reported protective or regulatory functions of γδ T cells in lung inflammation and fibrosis, including roles in tissue repair and resolution of inflammation (Pociask et al., 2011; Braun et al., 2008; Lo et al., 1950). These contrasting findings highlight the context-dependent nature of γδ T cell responses, which may vary according to disease stage, microenvironment, and cellular interactions.

Invariant natural killer T (iNKT) cells also respond to IL-23 by producing IL-17 and IL-22 (Michel et al., 2007). iNKT cells are abundant in the lung and act as bridges between innate and adaptive immunity. A growing body of evidence from both experimental models and human studies highlights the central role of iNKT cell activation in orchestrating inflammatory cascades that contribute to fibrotic diseases. Activation of iNKT cells has been shown to drive fibrosis-promoting mechanisms such as neutrophil recruitment (Michel et al., 2007), inflammasome activity (Kumar et al., 2023), and macrophages (Kim et al., 2008) and fibroblast activation (Syn et al., 2012). Recent findings further indicate that iNKT-associated pathways intersect with type 1, type 2, and type 3 immune responses, all of which are implicated in fibrotic processes such as IPF (Crosby and Kronenberg, 2018). These insights suggest that targeting upstream events—particularly the inhibition of iNKT cell activation—may represent a potential strategy for modulating diverse fibrotic pathways across multiple disease contexts.

### 1.3 Role of IL-23 in corticosteroid resistance

**FIGURE 2:**
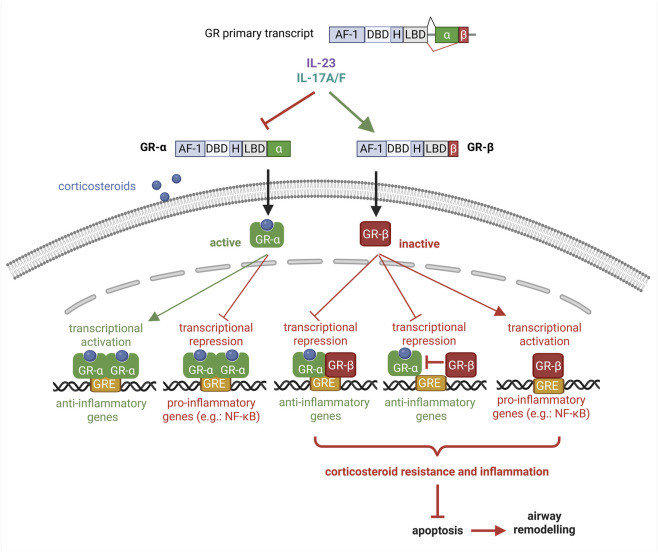
IL-23/IL-17–mediated regulation of glucocorticoid receptor signaling. IL-23 and IL-17A/F modulate alternative splicing of the glucocorticoid receptor (GR) primary transcript, suppressing GR-α and promoting GR-β expression. Corticosteroid-activated GR-α induces anti-inflammatory gene expression and represses proinflammatory pathways, whereas GR-β is transcriptionally inactive and antagonizes GR-α function. An increased GR-β/GR-α ratio results in corticosteroid resistance, persistent inflammation, reduced apoptosis, and airway remodeling.

The glucocorticoid receptor (GR) exists in multiple isoforms, generated mainly through alternative splicing and alternative translation initiation. The classical isoform, GR-α, binds glucocorticoids and translocates to the nucleus, where it activates anti-inflammatory genes and suppresses proinflammatory factors such as NF-κB (Ramos-Ramírez and Tliba, 2021; Huang and Wang, 2023) (Figure 2). In contrast, GR-β arises through alternative splicing at exon 9β and functions as a dominant-negative inhibitor: it can bind DNA response elements but lacks the ligand-binding domain, preventing glucocorticoid binding (Min et al., 2018) (Figure 2). Other isoforms, including GR-γ, GR-A, and GR-P, display lower transcriptional activity; however, their relative abundance can modulate an individual’s sensitivity to glucocorticoids.

A decreased GR-α/GR-β ratio is a hallmark of corticosteroid resistance and may result from upregulated GR-β expression, reduced GR-α expression, or both (Kelly et al., 2008) (Figure 2). Decreased corticosteroid sensitivity—especially in immune and epithelial cells—is often driven by inflammatory signals or oxidative stress. These factors can shift isoform balance toward GR-β or impair GR-α signaling through mechanisms such as phosphorylation, altered coregulator interactions, or reduced HDAC2 activity, thereby promoting resistance (Huang and Wang, 2023; Kelly et al., 2008).

Emerging evidence indicates that IL-23 plays a critical role in the development of corticosteroid resistance in lung inflammation by modulating both immune and structural cell functions. In peripheral blood mononuclear cells derived from patients with asthma, IL-23, in combination with IL-17, has been shown to upregulate GR-β expression and reduce the GR-α/GR-β ratio, thereby diminishing dexamethasone responsiveness and weakening the suppression of inflammatory genes (Vazquez-Tello et al., 2013) (Figure 2). In addition to its effects on immune cells, IL-23 and other Th17-related cytokines confer steroid resistance to airway structural cells, such as fibroblasts and endothelial cells, by inhibiting glucocorticoid-induced apoptosis. Upregulation of GR-β/GR-α ratio by IL-23/IL-17 could be associated with the relative corticosteroid-insensitivity of IPF (Lo et al., 2022), COPD (Nayak et al., 2026), and severe and treatment-refractory asthma (Halwani et al., 2016). The importance of IL-23 signaling in corticosteroid resistance has also been demonstrated *in vivo*. In murine models of asthma, epithelial IL-23 expression increases during allergen exposure, whereas pharmacological blockade of IL-23 leads to significant reductions in airway inflammation, mucus production, and Th2/Th17 cytokine levels—features that are closely associated with corticosteroid-resistant disease phenotypes (Lee et al., 2017). Moreover, in chronic and fibrotic lung disorders, elevated IL-17/IL-23 pathway activity has been correlated with increased GR-β expression and poor responsiveness to corticosteroids (Lo et al., 2022). Mechanistically, this resistance is amplified through downstream signaling involving STAT3, MAPK pathways, and reduced HDAC2 activity, all of which impair GR-α function and disrupt normal glucocorticoid signaling (Lo et al., 2022). Together, these findings highlight IL-23 as a key mediator of corticosteroid resistance across multiple cellular compartments and disease contexts, underscoring its potential as a therapeutic target in severe, treatment-refractory airway inflammation.

### 1.4 Overview of IL-23-targeted therapies

IL-23 is closely associated with inflammatory and autoimmune diseases such as rheumatoid arthritis (RA), systemic lupus erythematosus (SLE) and inflammatory bowel disease (Moschen et al., 2019; Abdo and Tye, 2020). While this clinical success has largely been established in autoimmune indications, it provides an important translational framework for evaluating IL-23 pathway modulation in lung and airway diseases. Serum IL-23 levels positively correlate with disease progression in RA (Melis et al., 2010) and ulcerative colitis (UC) (Mirsattari et al., 2012), therefore, targeting IL-23 and IL-23R is an effective strategy for treating autoimmune diseases (Xiong et al., 2022). IL-23 is also expressed in other disease contexts, including periodontal disease, ocular diseases, and cancers, further expanding the potential applications of IL-23-targeted therapy (Bunte and Beikler, 2019). The IL-23 inhibitors currently in clinical use include ustekinumab, guselkumab, risankizumab, tildrakizumab, and mirikizumab (Tables 2, 3).

**TABLE 2:** Approved IL-23 Inhibitors in Clinical Use (for detailed dosing, see Table 3).

Antibody	Target	Indications	Clinical efficacy	Unique features	References
Ustekinumab	p40 (shared by IL-12/23)	Moderate-to-severe plaque psoriasis; psoriatic arthritis; Crohn’s disease; ulcerative colitis	Higher PASI75 and PASI90 vs. placebo in psoriasis trials	First IL-23–inhibiting biologic approved by FDA (2009); broad efficacy across autoimmune diseases	Leonardi et al. (2008), Moschen et al. (2019), Wojno et al. (2019), Papp et al. (2013), Sands et al. (2019), Sands et al. (2022a), McInnes et al. (2013)
Guselkumab	p19 (IL-23 specific)	Moderate-to-severe psoriasis; psoriatic arthritis; Crohn’s disease; ulcerative colitis	Superior efficacy to ustekinumab in plaque psoriasis; sustained responses	First IL-23–specific inhibitor approved by FDA	Megna et al. (2018), Sbidian et al. (2021), Rahman et al. (2023), Wang et al. (2025b), Rubin et al. (2025)
Risankizumab	p19 (IL-23 specific)	Moderate-to-severe plaque psoriasis; psoriatic arthritis; Crohn’s disease; ulcerative colitis	Strong and durable improvement in skin and gut inflammation	Expanding applications across autoimmune disorders	Gordon et al. (2018), Östör et al. (2022), Feagan et al. (2017), Louis et al. (2024)
Tildrakizumab	p19 (IL-23 specific)	Moderate-to-severe plaque psoriasis	Significant reduction in disease severity and relapse rates	Well tolerated; long-lasting psoriasis control	Reich et al., 2017; Fala (2019)
Mirikizumab	p19 (IL-23 specific)	Crohn’s disease; ulcerative colitis	Improves clinical remission, endoscopic healing, quality of life; reduces bowel urgency	Gut-selective clinical focus	Sands et al. (2024), Sands et al. (2022b), D’Haens et al. (2023), Jairath et al. (2025), Lee et al. (2025)

**TABLE 3:** Approved IL-23 inhibitors: Dosage and frequency (IV-intravenous; SC-subcutaneous).

Antibody	Typical dosage	Dosing schedule	References
Ustekinumab	45–90 mg SC (psoriasis/PsA) ∼6 mg/kg IV (IBD induction)	Induction: weeks 0 and 4 Maintenance: every 8–12 weeks	(Drugs.com, 2025; Stelara ustekinumab, 2024)
Guselkumab	100 mg SC	Induction: weeks 0 and 4 Maintenance: every 8 weeks	(Johnson, 2024)
Risankizumab	150 mg SC (plus IV induction for IBD)	Induction: weeks 0 and 4 Maintenance: every 12 weeks	(U.S. FDA approves second indication, 2016; U.S. FDA approves SKYRIZI, 2024)
Tildrakizumab	100 mg SC	Induction: weeks 0 and 4 Maintenance: every 12 weeks	(FDA approves ILUMYA for treatment, 2019)
Mirikizumab	300 mg IV induction, then 200 mg SC	Induction: every 4 weeks Maintenance: SC every 4 weeks	(Drugs.com, 2024)

Ustekinumab was the first IL-23 inhibiting biologic approved by the Food and Drug Administration (FDA) for the treatment of moderate-to-severe plaque psoriasis in 2009 (Wojno et al., 2019; Schafer et al., 2010). In initial studies, ustekinumab-treated patients showed higher PASI75 and PASI90 response rates compared to placebo-treated controls (Leonardi et al., 2008; Papp et al., 2013). Ustekinumab has since also been approved for the treatment of Crohn’s disease (CD; 2016), UC (2019), and psoriatic arthritis (2013) (Moschen et al., 2019; Sands et al., 2019; Sands et al., 2022a; McInnes et al., 2013).

Guselkumab, the first IL-23-specific inhibitor targeting the p19 subunit, was approved by the FDA as a more effective treatment of moderate-to-severe plaque psoriasis than ustekinumab in 2017 (Megna et al., 2018; Sbidian et al., 2021). In 2020, it was also approved for the treatment of psoriatic arthritis (Rahman et al., 2023), and for UC and CD in 2024 (Wang H. et al., 2025; Rubin et al., 2025).

Risankizumab, another humanized IgG1 monoclonal antibody specific for the p19 subunit, has been approved by the FDA for the treatment of patients with moderate-to-severe plaque psoriasis (2019), psoriatic arthritis (2022), CD (2022), and UC (2023) (Gordon et al., 2018; Östör et al., 2022; Feagan et al., 2017; Louis et al., 2024).

Tildrakizumab has been approved by the FDA and European Medicines Agency for the treatment of moderate-to-severe plaque psoriasis in 2018 (Reich et al., 2017; Fala, 2019). Unlike other IL-23 inhibitors, tildrakizumab has not yet been approved for psoriatic arthritis, CD, or UC - though clinical trials are ongoing for other psoriatic conditions (e.g., plaque psoriasis: NCT04991116; genital psoriasis: NCT06029257; psoriasis: NCT05683015).

Mirikizumab is a fully humanized IgG4 variant monoclonal antibody that selectively binds to the p19 subunit of IL-23, approved for UC since 2023 and CD since 2025 (Steere et al., 2023). Mirikizumab provides strong, durable clinical and endoscopic remission (Sands et al., 2024; Sands et al., 2022b; D’Haens et al., 2023). Furthermore, it improves bowel urgency (Jairath et al., 2025) and quality of life (Lee et al., 2025), and offers a well-tolerated, targeted option for long-term inflammatory bowel disease management.

Given the central role of IL-23 in driving Th17 polarization and neutrophilic inflammation, it has become a critical target for therapeutic intervention. IL-23 has been studied extensively in autoimmune diseases, showing direct and indirect association with disease progression and severity. However, despite evidence indicating the involvement of the IL-23/Th17 axis in lung and airway diseases, as well as its association with disease severity and corticosteroid resistance (Rahmawati et al., 2021; Barnes, 2019), further research is required to clarify the direct role of IL-23 in the pathogenesis of lung diseases. Thus, in this review, we summarize current knowledge on the role of IL-23 in the pathogenesis of lung and airway diseases such as COPD, asthma, IPF, and COVID-19.

## 2 Role of IL-23 in pulmonary homeostasis and lung diseases

**FIGURE 3:**
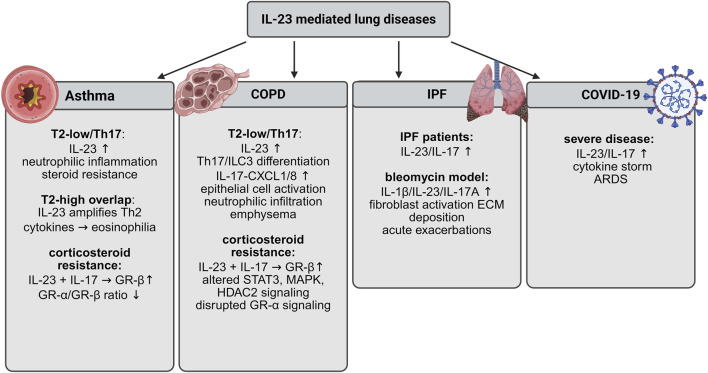
Comprehensive overview of the current understanding of IL-23–mediated mechanisms in asthma, COPD, IPF, and COVID-19. IL-23 promotes Th17/IL-17 responses, leading to neutrophilic inflammation, tissue remodeling, and disease severity. In asthma and COPD, IL-23 contributes to steroid resistance and chronic inflammation; in IPF, it drives fibroblast activation and fibrosis; and in severe COVID-19, elevated IL-23/IL-17 is associated with cytokine storm and ARDS.

Recent evidence indicates that the IL-23/Th17 axis is a critical regulator of both lung homeostasis and the immunopathogenesis of airway and parenchymal lung diseases. Elevated IL-23 expression and signaling are associated with greater disease severity, increased exacerbation frequency, reduced responsiveness to corticosteroid therapy, and airway remodeling, underscoring its pathogenic relevance. In the following section, we provide a comprehensive overview of IL-23–mediated mechanisms in pulmonary homeostasis and in the pathogenesis of asthma, COPD, idiopathic pulmonary fibrosis, and COVID-19 (Figure 3).

### 2.1 IL-23 in pulmonary homeostasis

IL-23–induced Th17 cytokines, particularly IL-17A and IL-22, play essential homeostatic roles in the lung by enhancing mucosal defense and preserving epithelial integrity (Dubin and Kolls, 2008; Ouyang and Valdez, 2008). In pulmonary epithelial cells, these cytokines induce antimicrobial peptides such as β-defensins and S100 proteins (e.g., S100A8/A9), which protect against pathogens including *Staphylococcus aureus* and *Klebsiella pneumoniae* (Aujla et al., 2008; Archer et al., 2016; Happel et al., 2005). Consistent with this function, mouse models of respiratory bacterial infection show that IL-23 deficiency compromises innate host defense, characterized by reduced neutrophil recruitment, attenuated Th17 and Th1 cytokine responses (including IL-17A and IFN-γ), and increased susceptibility to *Streptococcus pneumoniae* colonization and dissemination in the lung (Kim et al., 2013).

Beyond antimicrobial defense, IL-17A and IL-22 strengthen the epithelial barrier by upregulating tight junction proteins such as claudins and occludin, thereby limiting microbial translocation across the airway epithelium (Gurczynski and Moore, 2018; Alcorn, 2020; Hebert et al., 2020). In murine models of cryptococcal lung infection, IL-23 acting in concert with IL-22 reduces lung tissue damage and promotes barrier homeostasis, highlighting its role in balancing protective immunity with tissue preservation (Diniz-Lima et al., 2024). IL-23–dependent IL-22 further supports epithelial proliferation and repair following injury, as demonstrated in influenza- and bleomycin-induced lung damage models (Pociask et al., 2013; Zhang et al., 2024). These protective effects are mediated largely through STAT3 and C/EBPβ signaling pathways, which regulate genes involved in antimicrobial activity, barrier maintenance, and epithelial regeneration (Aujla et al., 2008).

In parallel, IL-17A downstream of IL-23 promotes homeostatic neutrophil recruitment and local granulopoiesis, enabling rapid containment of inhaled pathogens without excessive immunopathology (Stark et al., 2005). Collectively, these functions position IL-23 as a central regulator of immune vigilance and tissue protection at the air–liquid interface of the respiratory tract. Under steady-state conditions, IL-23 sustains a controlled type 17 immune tone that supports barrier integrity, microbial containment, and lung resilience. However, disruption of this homeostatic axis—such as in asthma, COPD, viral infections, or pulmonary fibrosis—can drive sustained IL-23/IL-17A signaling and contribute to chronic inflammation and immunopathology.

### 2.2 Asthma

Asthma is a chronic inflammatory airway disease, characterized by reversible airflow obstruction, airway hyperresponsiveness (AHR), remodeling, and enhanced mucus production. Phenotypes, representing the clinical presentation, are broadly classified into allergic and non-allergic, whereas the endotypes, reflecting underlying molecular mechanisms, are classified into T2-high and non-T2/T2-low asthma (Agache and Akdis, 2016). T2-high allergic asthma is the most common asthma phenotype, typically driven by sensitization to aeroallergens such as house dust mites, pollen, or fungal spores. This T2-high endotype is generally responsive to corticosteroid therapy (Agache and Akdis, 2016).

#### 2.2.1 Clinical asthma endotypes and human evidence for IL-23 involvement

Severe corticosteroid-resistant asthma has been divided into two inflammatory subtypes: the high eosinophil/high neutrophil group and the low eosinophil/high neutrophil group (Nakagome et al., 2012). This classification underscores marked heterogeneity in severe asthma, with concurrent eosinophilic and neutrophilic inflammation of the airways (Nakagome et al., 2012). IL-23 levels are significantly higher in the sputum of patients with severe asthma compared to those with milder asthma (Hastie et al., 2018). Severe asthma has also been associated with overexpression of Th17 cytokines, increased numbers of IL-23-positive cells and neutrophils in the bronchial mucosa (Ricciardolo et al., 2017), further supporting a contributory role for IL-23 in asthma exacerbation.

With increasing disease severity, accumulating evidence implicates IL-23 as a mediator across both T2-high and non-T2 endotypes (Wu and Peebles, 2023). IL-23 is elevated in eosinophilic asthma, whereas IL-17A is more closely associated with allergic asthma and shows a positive correlation with FeNO levels (Domvri et al., 2019). Moreover, elevated IL-23 levels inversely correlate with forced expiratory volume in the first second (FEV1) in asthmatic children (Ciprandi et al., 2012a) and are associated with increases oxidative stress in patients with non-T2 work-related asthma and silicosis (Kurt et al., 2023). Together, these clinical and biomarker-based observations position IL-23 at the interface between asthma endotype heterogeneity, disease severity, and impaired lung function.

#### 2.2.2 Upstream induction of IL-23 and downstream molecular mechanisms in asthma

In the T2-low and T2-high/T17-high endotypes, exposure to external stimuli such as polluted air, cigarette smoke, and diesel exhaust particles initiates an IL-23–JAK2/TYK2–STAT3–Th17 signaling cascade, resulting in sustained airway inflammation (Brandt et al., 2013; Polosa and Thomson, 2013). In the presence of cytokines such as IL-6, IL-1β, and TGF-β, IL-23 supports Th17 differentiation and polarization from naïve T cells, leading to the release of Th17 cytokines, including IL-17A, IL-17F, and IL-22 (McGeachy et al., 2009). Mechanistically, IL-23-driven STAT3 activation enhances RORγt expression and maintains pathogenic Th17 cell survival, thereby sustaining neutrophilic airway inflammation and epithelial activation (Nightingale, 2020). IL-23 also activates ILC3 to release IL-17. Together, Th17 and ILC3 cells (Th17 endotype) enhance neutrophil activation and migration, resulting in neutrophilic inflammation (Agache and Akdis, 2016; Boonpiyathad et al., 2019). Downstream, IL-17A/F induce epithelial chemokines (e.g., CXCL8/IL-8), directly linking IL-23 signaling to airway neutrophilia and hyperresponsiveness (Lubberts, 2015; Al-Ramli et al., 2009).

In eosinophilic asthma, IL-23 sustains Th17 cells and IL-17A/F production, which synergize with Th2 cytokines (IL-4, IL-5, IL-13) to induce epithelial eotaxins (CCL11/24/26), GM-CSF, and alarmins (TSLP, IL-33), thereby amplifying eosinophil recruitment, survival, corticosteroid resistance, and airway remodeling in severe eosinophilic asthma (Wu and Peebles, 2023; Nakajima and Hirose, 2010; Wang et al., 2010). Further, IL-23 has been shown to significantly inhibit dexamethasone-induced apoptosis of cultured airway fibroblasts and endothelial cells, which may contribute to asthma pathogenesis (Halwani et al., 2016). Mechanistically, this effect is mediated by STAT3/MAPK-dependent survival signaling in airway structural cells, linking IL-23 to corticosteroid resistance and airway remodeling. Therefore, this Th17 endotype associates with neutrophilic airway inflammation, a common feature in corticosteroid-resistant asthma and severe asthma (Li and Hua, 2014).

#### 2.2.3 Causal evidence from experimental asthma models

Causal involvement of IL-23 in asthma pathogenesis has been demonstrated in multiple experimental models. In mice challenged with house dust mite extract, a significant increase in IL-23 and IL-23R-positive macrophages was observed, accompanied by enhanced IL-17 production and neutrophil recruitment (Leitner et al., 2022). Intranasal administration of IL-23 together with diesel exhaust particles led to increased eosinophilic inflammation and bronchial hyperresponsiveness to methacholine, both characteristic features of non-allergic eosinophilic asthma (Lee et al., 2020). Similarly, IL-23 enhanced antigen-induced activation of both Th17 and Th2 cells, thereby increasing Th2-mediated eosinophil recruitment and Th17-mediated neutrophil recruitment into the airways (Wakashin et al., 2008). In mouse models of OVA- and *A*. *fumigatus-*induced airway inflammation, 95% of IL-23p19^+^ cells were eosinophils, suggesting that eosinophils contribute to the immunomodulation of the IL-23/Th17 axis in addition to their role as effector cells of inflammation (Guerra et al., 2017).

IL-23 knockdown reduced the numbers of eosinophils and neutrophils in the bronchoalveolar lavage of mice with allergic airway inflammation, along with a significant decrease of IgE, IL-17, and IL-4 in the serum (Li et al., 2011). Overexpression of IL-23R in T cells led to increased pulmonary eosinophil infiltration in ovalbumin-induced mouse models of airway inflammation compared to wild-type controls. Additionally, *ex vivo* stimulation of IL-23R-overexpressing T-cells resulted in higher levels of Th2 cytokines (Peng et al., 2010). A recent study confirmed that even LPS-induced inflammation in mice is associated with a significant increase in IL-23 expression in macrophages (Holbrook et al., 2023). Another mouse airway neutrophilia model showed that exogenous IL-25 reduced neutrophil counts in the bronchoalveolar lavage by suppressing IL-23 (Chang et al., 2023). These gain- and loss-of-function experiments (IL-23 administration, IL-23 knockdown, and IL-23R overexpression) provide direct causal evidence that IL-23 is sufficient and necessary to drive mixed Th2/Th17 airway inflammation and AHR in experimental asthma.

#### 2.2.4 Pharmacological targeting of the IL-23 axis in asthma

Pharmacological blockade of IL-23 with monoclonal antibodies reduced airway inflammation in multiple murine models, providing direct interventional evidence for an upstream pathogenic role of IL-23. For instance, administration of anti-IL-23p19 mAb in OVA-induced mice reduced IL-23/Th17 cytokines such as IL-23p19 and IL-17A in the airways. This treatment also decreased inflammatory cell recruitment, IL-4 in BALF, and mucus secretion. The results from this study indicate that IL-23 is essential for maintaining the Th17 axis and allergic inflammation in the OVA-induced model (Zhang et al., 2020). Moreover, treatment with anti-IL-23 antibodies reduced immune cell infiltration in BALF, AHR, and mucus secretion in an OVA-induced mouse model of allergic airway inflammation, along with decreased IL-17 levels and Tc17 cells in lung tissue homogenate (Cheng et al., 2016; Ogawa et al., 2015). Similarly, a study by Bruggemann et al. demonstrated that targeting IL-23, either with a monoclonal antibody against IL-23p19 (αIL-23) or with the IL-23-binding protein anticalin-3, in a mouse model of allergic lung inflammation with a mixed granulocytic phenotype significantly reduced macrophage accumulation, IL-17^+^ CD4^+^ T cells, and AHR (Brüggemann et al., 2024). Increased IL-23 and IL-23R expression in lung epithelium and ILC2 cells was reported in CS (cigarette smoke)-induced allergen-sensitized mice, which when treated with an anti-IL-23p19 antibody showed reduced allergic airway inflammation, providing early evidence that IL-23 blockade can attenuate asthma-associated pathology suggesting that the IL-23 pathway may serve as a pharmacological target in allergen-induced asthma (Lee et al., 2019). Further, IL-23 was significantly elevated in the lung tissues of neonatal mice exposed to particulate matter and house dust mites, promoting AHR and increased eosinophil and neutrophil levels. This effect was mitigated by using an IL-23 blocking monoclonal antibody during early exposure, clearly indicating an important role of IL-23 in particulate matter-induced asthma in early life (Park and Lee, 2024).

Notably, a macrophage–IL-23–neutrophil causal axis has been identified in neutrophilic asthma. Using a neutrophil-dominant asthma model, Han and colleagues identified a unique subset of CD39^+^CD9^+^ interstitial macrophages that suppress neutrophil extracellular trap formation and inflammation (Han et al., 2024). However, IL-23 was found to inhibit these macrophages, an effect that could be reversed by treatment with an IL-23 inhibitor (αIL-23p19), demonstrating that IL-23 causally disrupts endogenous anti-inflammatory macrophage programs and promotes neutrophilic inflammation (Han et al., 2024).

Taken together, these preclinical data implicate IL-23 as a key upstream modulator of mixed granulocytic airway inflammation across asthma endotypes, providing a rationale for therapeutic targeting of the IL-23 axis.

#### 2.2.5 Therapeutic implications and translational perspectives for asthma

There is currently no permanent cure for asthma. Existing treatments focus primarily on prevention and long-term control. Common therapies include inhaled corticosteroids, leukotriene modifiers, beta mimetics, muscarinic antagonists, and biologics (Castillo et al., 2017). Although effective, these therapies have limitations such as side effects, high costs, and variable patient responses. As a result, multiple approaches are often required for effective management, particularly in severe non-T2 asthma with corticosteroid resistance.

As summarized above, several studies have highlighted the beneficial effects of anti-IL-23 antibodies in experimental models of airway inflammation. However, the anti-interleukin-23p19 monoclonal antibody risankizumab failed to show clinical efficacy in patients with severe asthma (Brightling et al., 2021). In addition, systemic and inhaled JAK inhibitors targeting the downstream signaling of IL-23 have been evaluated in preclinical and clinical studies (Georas et al., 2021; Luschnig et al., 2021). GDC-0214, an inhaled JAK1/JAK2 inhibitor, reduced airway inflammation with good safety in a Phase 1 asthma trial (Braithwaite et al., 2021). Frevecitinib (KN-002; NCT05006521), an inhaled pan-JAK inhibitor, was well tolerated and caused a clinically relevant reduction of fractional exhaled nitric oxide in patients with moderate-to-severe asthma and is advancing to Phase 2b (Singh et al., 2025). These mixed clinical outcomes emphasize that effective targeting of the IL-23 pathway will likely require biomarker-driven patient selection and a refined understanding of disease endotypes.

### 2.3 Chronic obstructive pulmonary disease

COPD is a chronic inflammatory lung disease, encompassing both chronic bronchitis and emphysema, associated with progressive, irreversible airflow obstruction. The FEV_1_/FVC ratio (ratio of the forced expiratory volume in the first second to the forced vital capacity of the lungs) in COPD is usually <70%, reflecting an obstructive spirometry pattern. According to the Global Initiative for Chronic Obstructive Lung Disease (GOLD), COPD is classified in four stages from 1-mild to 4-very severe based on the degree of airflow limitation (GOLD Report. Global Initiative for Chronic, 2024).

COPD presents with multiple clinical phenotypes, reflecting substantial heterogeneity in disease severity and progression. Neutrophilic inflammation is a characteristic feature of COPD, reflected by increased neutrophil counts in the blood and sputum. Notably, this neutrophilic inflammatory endotype has been associated with corticosteroid resistance (Barnes, 2019). A subset of COPD patients also exhibits eosinophilic inflammation, indicated by increased eosinophil levels in sputum and blood. Eosinophilia in COPD has been considered an overlapping feature with asthma, associated with increased IL-5 levels (Barnes, 2016a). Moreover, high blood eosinophil levels serve as a biomarker for predicting the response to inhaled corticosteroids in acute exacerbations of COPD. Disease progression is characterized by recurrent exacerbations and is frequently associated with respiratory infections.

#### 2.3.1 COPD severity and human evidence for IL-23 involvement

Lung inflammation and formation of emphysema are believed to be regulated, at least in part, by the IL-23/Th17 pathway (Fujii et al., 2016). In COPD patients, higher serum concentrations of IL-23 have been reported (Rong et al., 2020). Serum IL-23 levels positively correlate with GOLD grading, mMRC score (Modified Medical Research Council), and longer clinical medical history, while showing a negative correlation with FEV1/FVC and FEV1% (Rong et al., 2020). IL-23 in sputum shows a significant correlation with the percentage of sputum neutrophils in healthy individuals and COPD patients and IL-23 expression is higher in patients with neutrophilic inflammation compared to those with eosinophilic airway inflammation (Moermans et al., 2021). Another study reported an increase in IL-23-positive cells in the bronchial epithelium and submucosa of COPD patients compared to non-smokers, suggesting that elevated IL-23 expression in the bronchial mucosa of stable COPD patients may contribute to disease pathogenesis (Di Stefano et al., 2009). Moreover, in patients with acute *Pseudomonas aeruginosa* exacerbations, bronchoalveolar IL-23 and IL-17A levels were significantly elevated compared with stable COPD, indicating upregulation of the IL-23/IL-17 axis in infected lungs (Ding et al., 2021). These data place IL-23 at the intersection of airway neutrophilia, emphysematous remodeling, and clinical decline in COPD.

#### 2.3.2 Upstream induction of IL-23 and downstream molecular mechanisms in COPD

In COPD, APCs such as macrophages and dendritic cells are particularly activated by cigarette smoke, oxidative stress, and microorganisms (Barnes, 2019; Vassallo et al., 2010). APC-derived IL-23 then binds to its receptor on T helper cells and promotes Th17 polarization and ILC3 stabilization (Rouzic et al., 2017). As recently demonstrated, cigarette smoke particularly shifts lung DC subsets (↓cDC1, relative ↑cDC2), thereby enhancing IL-6/IL-23-driven Th17 polarization and promoting Th17/Treg imbalance (Mengistu et al., 2026). TGF-β upregulates IL-23R expression, thereby contributing to the differentiation process (Mangan et al., 2006), whereby IL-23 primarily supports Th17 cell expansion, survival, and maintenance (Rouzic et al., 2017). Th17 cells release IL-17, which further activates epithelial cells, inducing the release of neutrophil-attracting chemokines such as CXCL1 and CXCL8/IL-8, thereby maintaining and recruiting neutrophils into the airways (Rouzic et al., 2017). Besides NF-kB and MAPK (Barnes, 2016b), the JAK-STAT signaling pathway critically contributes to these mechanisms, as IL-23 signals via JAK2-TYK2, leading to STAT3/STAT4-dependent Th17 polarization (Alcorn et al., 2010). Moreover, IL-23 has been linked with mucus production in COPD. In Mucin-5B (Muc5b) −/− mice, apoptotic macrophages accumulated in the lungs and IL-23 production was reduced (Roy et al., 2014), whereas in Muc5b transgenic mice, IL-23 production increased along with enhanced macrophage function (Roy et al., 2014). IL-23 thus converts innate danger sensing into chronic neutrophilic airway inflammation in COPD.

#### 2.3.3 Causal evidence from experimental *in vitro* and *in vivo* models

In a mouse model, chronic cigarette smoke (CS) exposure increased lung Th17 cells, RORγt, and IL-17A/IL-6/IL-23, while reducing Tregs, Foxp3, and IL-10 compared with sub-acute exposure and air controls, with a parallel increase in the Th17/Treg ratio in peripheral blood (Wang et al., 2012). Liang and colleagues demonstrated that IL-23 produced by activated dendritic cells in response to cigarette smoke exposure promotes differentiation of innate lymphoid cells into natural cytotoxicity receptor-negative (NCR-) ILC3s, which secrete IL-17A, sustain neutrophilic inflammation, and contribute to chronic lung inflammation. This IL-23/ILC3 axis represents a potential mechanism sustaining immune dysregulation and tissue damage in smoking-related COPD (Liang et al., 2024). In an elastase/LPS-injured mouse model, Respiratory Syncytial Virus (RSV) infection amplified neutrophilic inflammation, IL-17/IL-23 signaling, mucus cell hyperplasia, and airspace enlargement. IL-17 depletion attenuated these RSV-driven pathological changes (Mebratu and Tesfaigzi, 2018). *P. aeruginosa* infection similarly increased IL-23/IL-17 signaling and drove neutrophilic inflammation and lung dysfunction, while IL-17A blockade reduced bacterial burden and pulmonary pathology in a COPD mouse model (Ding et al., 2021). By contrast, in a COPD model complicated by invasive pulmonary aspergillosis (IPA), Th17/IL-17 responses were dysregulated compared with COPD or IPA alone. Genetic deletion of IL-17 increased fungal burden, indicating a protective antifungal role for IL-17 in COPD + IPA (Geng et al., 2020). Similarly, in a cigarette smoke (CS)-induced murine model with *S*. *pneumoniae* infection, defective production of the protective Th17 cytokine IL-22 by conventional T cells (Pichavant et al., 2015) was observed, while exogenous administration of IL-22 protected CS-exposed mice from bacterial infection. This effect was accompanied by impaired IL-23 production in alveolar macrophages and dendritic cells of CS-exposed mice. Consistently, in COPD patients, Th17 cytokine (IL-22, IL-17A) responses to bacterial infection were also impaired, which was associated with reduced IL-23 production from peripheral blood mononuclear cells (PBMCs) (Pichavant et al., 2015). Together, genetic and interventional studies demonstrate a causal role for IL-23–dependent Th17/ILC3 polarization in driving neutrophilic airway inflammation and emphysematous remodeling in COPD, while also revealing a context-dependent protective role of IL-23/IL-17 signaling in antimicrobial host defense.

#### 2.3.4 Therapeutic implications and translational perspectives for COPD

Currently, there is no cure for COPD. Available treatments, including bronchodilators, corticosteroids, and antibiotics, alleviate symptoms but do not address the underlying disease mechanisms (Vogelmeier et al., 2017; Luschnig et al., 2021). Given the established role of IL-23 in the pathogenesis and corticosteroid resistance in COPD, it represents a potential therapeutic target for the development of precision medicines. Accordingly, recent work has shown that blocking IL-23 signaling with a monoclonal antibody in a murine emphysema model significantly reduced smoke-induced emphysema along with immune cell infiltration, oxidative stress, and apoptosis (Tian et al., 2024). Similarly, IL-17-neutralizing antibody treatment was effective in a CS-induced model even after lung damage had been established (Riani Moreira et al., 2025). A study by Mardi et al. reported that in patients with moderate-to-severe COPD, nanocurcumin supplementation significantly reduced IL-23 levels, concomitantly downregulating Th17 cells and proinflammatory cytokines such as IL-17, resulting in a reduction in systemic inflammation, highlighting the significance of targeting IL-23 in COPD patients (Mardi et al., 2024). In addition, preclinical studies demonstrate that JAK inhibitors can reduce lung inflammation and tissue damage in COPD models (Fenwick et al., 2015; Milara et al., 2022). Furthermore, inhaled frevecitinib (KN-002; NCT05006521) was well tolerated in COPD patients, supporting further clinical evaluation of pathway-targeted interventions (Morgan et al., 2024). Together, these converging preclinical and early clinical signals argue for repositioning IL-23-pathway modulation from broad immunosuppression toward endotype-informed intervention aimed at interrupting the inflammatory circuits that drive COPD progression.

### 2.4 Idiopathic pulmonary fibrosis

Idiopathic pulmonary fibrosis is a progressive interstitial lung disease affecting 3 million people worldwide, with a median survival of 3–5 years if untreated (Nalysnyk et al., 2012; Martinez et al., 2017; Lederer and Martinez, 2018). It is characterized by excessive fibrotic tissue and extracellular matrix (ECM) deposition, which compromises alveolar structure and gas exchange (Sgalla et al., 2018). Risk factors include smoking, dust exposure, infections, and genetic predisposition (Martinez et al., 2017). In 2014, the FDA approved nintedanib and pirfenidone, which slow lung function decline but are not curative (Sgalla et al., 2018).

Unlike asthma, COPD, and COVID-19, IPF is a restrictive disease originating from repetitive epithelial damage and aberrant repair processes. Notably, several studies have associated inflammatory dysregulation and inflammatory cytokines with the outcome of the disease, disease stage, lung cellular physiology, and disease severity (She et al., 2021; Tomos et al., 2016). Alveolar type I (ATI) cells enable gas exchange, while type II (ATII) cells produce surfactant and regenerate ATI cells (Guillot et al., 2013). Persistent epithelial damage triggers the secretion of fibrotic mediators such as TGF-β, PDGF, and TNF-α, promoting fibroblast and myofibroblast expansion and further ECM accumulation (Klingberg et al., 2013; Selman and Pardo, 2012; Moore and Herzog, 2013). The immune system also contributes, though its role remains unclear. Macrophages promote ECM turnover and fibroblast proliferation via TGF-β (Ogawa et al., 2021; Zhang et al., 2018; Jegal, 2022). Neutrophils release cytokines, neutrophil elastase, and extracellular traps, worsening injury and altering ECM dynamics (Suzuki et al., 2019; Chua et al., 2007).

#### 2.4.1 Human evidence for IL-23 involvement in IPF

Th2/Th17 cells drive fibrosis through IL-4, IL-13, and IL-17, while Th1 cells may counteract fibrotic processes (Keane et al., 2001; Saito et al., 2003; Nie et al., 2022; Prior and Haslam, 1992). Although causality remains to be established, available human data point to increased Th17-associated cytokine expression in IPF. Zhang et al. observed that IL-22, IL-23, and IL-17 were significantly increased in the serum of lung cancer patients with IPF compared to healthy controls and lung cancer patients without IPF (Zhang et al., 2022). Furthermore, IL-1β and IL-17A are elevated in the BALF of patients with IPF (Wilson et al., 2010), whereby IL-17 further increases during acute exacerbations (Weng et al., 2019). In addition to airway fluid and serum findings, IL-17 expression is increased in fibrotic lung tissue from IPF patients, with higher numbers of IL-17+ cells detected by immunohistochemistry, and immune profiling of patient samples demonstrates expanded IL-17A–producing CD4^+^ T-cell subsets, supporting heightened IL-17 pathway activity in human IPF (Wilson et al., 2010; Lo et al., 2022). Overall, the available human evidence supports a contributory role for IL-23/IL-17–driven inflammation in the pathogenesis and progression of IPF.

#### 2.4.2 Upstream induction of IL-23 and downstream molecular mechanisms in IPF

At the molecular level, IL-23 exerts its profibrotic effects in the lung predominantly through the IL-23/IL-17/IL-22 axis, linking chronic inflammation to irreversible tissue remodeling. In response to repetitive epithelial injury, activated macrophages and dendritic cells secrete IL-23, which promotes the survival and expansion of pathogenic Th17, γδ T, and ILC3 cells. These cells release downstream cytokines such as IL-17 and IL-22, which stimulate TGF-β1 production and induce the epithelial-mesenchymal transition in alveolar type I (ATI) and type II (ATII) cells involving impaired mitochondrial homeostasis (Xiao et al., 2022) as well as Smad2/3 and ERK1/2 signaling (Wang et al., 2017). This molecular reprogramming causes healthy epithelial cells to lose their polarity and transform into mesenchymal-like cells that produce excessive extracellular matrix. Concurrently, IL-23 may enhance fibroblast recruitment and accumulation of myofibroblasts, including through CCR2-associated pathways. Moreover, upregulation of GR-β by IL-17 could be associated with the relative corticosteroid-insensitivity of IPF (Lo et al., 2022). Together, these mechanisms position IL-23–driven IL-17/IL-22 signaling as a central driver of fibrotic remodeling and treatment resistance in the IPF lung.

#### 2.4.3 Causal evidence from experimental IPF models

The current knowledge regarding the role of IL-23 in the pathogenesis of IPF has been primarily derived from the murine bleomycin model of pulmonary fibrosis. This model involves the intratracheal administration of bleomycin, a chemotherapeutic agent that induces fibrotic remodeling of the lungs. A study by Gasse et al. showed that bleomycin exposure increases IL-1β, which drives IL-23p19 and IL-17A production within 24 h. In this setting, γδ T cells and Th17 cells have been identified as early cellular sources of IL-17. Treatment with IL-1β alone produced similar effects, highlighting an IL-1β/IL-23/IL-17A axis in early bleomycin-induced pulmonary fibrosis. Neutrophils exacerbate inflammation, and IL-23p19-deficient mice displayed reduced neutrophil recruitment, myeloperoxidase activity, and absence of fibrosis-related mediators such as TIMP-2 compared to controls (Gasse et al., 2011). Moreover, genetic deficiency or blockade of IL-23p19 or IL-17 signaling reduces TGF-β1 production and collagen deposition, indicating that this axis is required for fibrotic remodeling in this model (Gasse et al., 2011). Notably, the development of acute exacerbation (AE) of pulmonary fibrosis has also been linked to IL-23. In a bleomycin/LPS-induced murine model of AE of pulmonary fibrosis, a dramatic increase in neutrophils, proinflammatory cytokines such as IL-6, TNF-α, and keratinocyte chemoattractant, as well as IL-23, IL-17A, and IL-22 was observed (Senoo et al., 2021). Interestingly, this inflammatory profile and the development of AE were absent in IL-23 knockout mice (Senoo et al., 2021). Further studies in knockout mice demonstrated that IL-17A is a central driver of inflammation and collagen deposition, with this profibrotic pathway promoted by IL-12/23p40, IFN-γ, and TGF-β, and counterregulated by IL-10 (Wilson et al., 2010). These findings support a causal role for IL-23 signaling in fibrosis development and in inflammatory amplification during experimental acute exacerbation.

While the above-discussed studies suggest that IL-23 influences the inflammatory profile during the onset and progression of fibrosis, a study by Zhang et al. highlights the potential of IL-23 in driving the fate of ATI cells during the onset of fibrosis in the context of rheumatoid-arthritis-associated pulmonary fibrosis (Zhang et al., 2021). ATI cells treated with IL-23 showed increased expression of mesenchymal markers such as αSMA, collagen type I and III, indicating a transition towards a mesenchymal phenotype under mechanical stress, compared to ATI cells retaining a fully epithelial phenotype (Zhang et al., 2021). Moreover, IL-23 increased the resistance of ATI cells to apoptosis, while increasing their invasive properties *in vitro* (Zhang et al., 2021)*.* Collectively, IL-23p19 knockout, IL-12/23p40 deficiency, and antibody-mediated blockade experiments establish a causal role for IL-23–IL-17 signaling in promoting TGF-β1 induction, collagen deposition, and fibrotic remodeling in experimental pulmonary fibrosis.

#### 2.4.4 Pharmacological targeting of the IL-23 axis in IPF

In line with previous findings, pharmacological inhibition of IL-23 with anti-IL-12/23p40 monoclonal antibodies in bleomycin/LPS-treated mice abrogated the onset of airway inflammation as well as the consequent pulmonary fibrosis (Senoo et al., 2021). Gasse et al. also showed that neutralizing IL-17A reduces TGF-β1 levels and attenuates airway inflammation and fibrosis in a murine bleomycin model (Gasse et al., 2011). Furthermore, a bispecific antibody targeting IL-17A/IL-1β reduced dermis thickening and lung fibrosis in a bleomycin-induced model of systemic sclerosis (Yin et al., 2020). In addition, targeting IL-23 signaling with JAK inhibitors such as baricitinib (Liu et al., 2023; Gu et al., 2023), filgotinib (Lv et al., 2025), or ruxolitinib (Bellamri et al., 2023) also shows beneficial effects in experimental models of lung fibrosis. While no IL-23–targeting therapy has been approved for IPF to date and no interventional clinical trials have evaluated anti–IL-23 or anti–IL-17 monoclonal antibodies in IPF, a case report suggests that IL-23 inhibition with guselkumab may stabilize lung function in patients with systemic sclerosis–associated interstitial lung disease (Fukasawa et al., 2024). Taken together, these studies suggest a potentially profibrotic role of IL-23 in pulmonary fibrosis, orchestrating both inflammatory and fibrotic processes, likely via IL-17–associated signaling. Further studies in animal models and patient samples are needed to define the impact of IL-23–mediated pathways on fibroproliferation, myofibroblast differentiation, and ECM biosynthesis, and to assess their therapeutic relevance.

### 2.5 Coronavirus disease 2019

Since the COVID-19 pandemic, several studies have focused on the cytokine landscape associated with the disease. In particular, severe COVID-19 is characterized by weak interferon responses and an excessive rise in proinflammatory cytokines (‘cytokine storm’), driving inflammation and predicting worse outcomes.

The pathophysiology of COVID-19 involves a dynamic interplay of viral invasion and immune dysregulation. SARS-CoV-2 infects cells through its spike protein, which binds to the ACE2 receptor, abundantly expressed in the respiratory tract and other organs (Parasher, 2020). This process is facilitated by proteases like TMPRSS2, enabling viral entry via endocytosis (Attaway et al., 2021). As an early immune response Toll-like receptors detect viral components, triggering the release of Type I/III interferons (Attaway et al., 2021). In COVID-19, this early interferon response is weak and delayed, allowing uncontrolled viral replication. As the infection progresses, immune cells, primarily macrophages and T cells, become overactivated, producing excessive proinflammatory cytokines such as IL-6, IL-1β, and TNF-α. This leads to a cytokine storm, a hyperinflammatory state that causes widespread tissue damage. In severe cases, ARDS develops as a result of damage to alveolar cells and endothelial barriers (Attaway et al., 2021), leading to fluid leakage into the lungs, impaired gas exchange, and hypoxemia. Additionally, endothelial dysfunction induced by SARS-CoV-2 contributes to thrombosis and capillary leakage (Wiersinga et al., 2020). Beyond the lungs, COVID-19 has systemic effects. The virus can directly or indirectly damage the heart, causing myocarditis, arrhythmias, and thrombosis. Similarly, acute kidney and liver injuries may arise from direct viral effects, hypoxia, and inflammation, illustrating the multisystemic impact of the disease (Wiersinga et al., 2020).

#### 2.5.1 Human evidence for IL-23 involvement in COVID-19

A growing body of clinical evidence indicates that IL-23 is dysregulated in COVID-19 and may be linked to disease severity and inflammatory responses. In a recent study, patients with COVID-19 showed a significant increase in serum levels of IL-23 compared to healthy controls, with even higher levels observed in critical cases compared to mild or severe cases (Smail et al., 2023). This observation was further confirmed by Markovic et al., who found increased serum concentrations of IL-23 correlating with disease severity (Markovic et al., 2021). IL-23, along with other cytokines such as IL-12, IFN-γ, and IL-17, was elevated in stage IV of the disease (Jovanovic et al., 2023). In addition, serum IL-23, together with IL-10 and TNF-α, was significantly higher in critical versus mild and severe COVID-19 and independently associated with worse in-hospital outcomes and mortality, with positive correlations between IL-23 and CRP levels (Smail et al., 2023). However, another study in 30 COVID-19 patients and 30 healthy controls did not find any disease-induced changes in IL-23, but revealed sex differences in IL-23, with male patients showing higher IL-23 serum levels than females (Abbasifard et al., 2022). Consistent with this heterogeneity, a study comparing critically ill COVID-19 patients with critically ill non-COVID-19 ICU patients of similar illness severity reported higher circulating IL-17 in COVID-19, while IL-23 levels were comparable between groups, and IL-17/IL-23 were undetectable in non-critically ill COVID-19 patients (Jahaj et al., 2021). IL-23 is also involved in modulating the gastrointestinal immune system in COVID-19, as increased faecal levels have been found in patients with severe COVID-19 (Britton et al., 2021). Serum IL-17 levels have also been consistently reported to be higher in COVID-19 patients compared to healthy controls, particularly in those with severe or critical illness, and IL-17A genetic associations further support its link to disease severity (Sadeghi et al., 2021; Ghazavi et al., 2021). Notably, some human studies suggest that not all IL-17 family members behave similarly in COVID-19 severity, with reports of IL-17F (rather than IL-17A) being elevated in severe disease (Bédard-Matteau et al., 2024). Overall, these human studies suggest that IL-23 is frequently elevated in severe COVID-19 and associates with proinflammatory cytokine profiles and adverse outcomes, although findings are heterogeneous and may be influenced by patient population, disease stage, and sex differences.

#### 2.5.2 Upstream induction of IL-23 and downstream molecular mechanisms in COVID-19

In COVID-19, increased IL-23/IL-17 levels are accompanied by increased expression of Th1 and Th17 transcriptional factors such as Tbet and RORγt in CD4^+^ cells, along with an increased percentage of IFN-γ and IL-17-producing T cells (Jovanovic et al., 2023). In addition, elevated serum IL-23 has been correlated with markers of inflammasome activation (e.g., IL-18, caspase-1 activity) and neutrophil recruitment signatures in critically ill patients, suggesting that innate cytokine networks contribute to IL-23 elevation and downstream Th17 skewing. In line with that, genetic data support a causal link between high TYK2 expression and life-threatening COVID-19 (Pairo-Castineira et al., 2021). Furthermore, functional genetic variation in IL23R and related JAK-STAT signaling genes has been associated with differential cytokine responses and disease severity in infection and inflammatory conditions, reinforcing the role of IL-23 pathway signaling in shaping adaptive immunity (Goda et al., 2023). The convergence of cytokine profiling, immune-cell transcriptional reprogramming, and TYK2 genetic evidence supports a mechanistic link between IL-23 pathway activation and pathogenic Th1/Th17 polarization in severe COVID-19, while underscoring the need to preserve protective antiviral immunity.

#### 2.5.3 Pharmacological targeting of the IL-23 axis in COVID-19

From a therapeutic point of view, baricitinib, a JAK1/2 inhibitor, is recommended by the WHO for hospitalized patients with severe or critical COVID-19 requiring oxygen, in addition to corticosteroids. Randomized trials demonstrated improved outcomes: COV-BARRIER showed reduced 28- and 60-day mortality (Marconi et al., 2021), RECOVERY confirmed a mortality benefit (Recovery Collaborative Group, 2022), and ACTT-2 reported faster recovery with baricitinib plus remdesivir (Kalil et al., 2021). Safety analyses found no increase in serious infections or thromboembolism, supporting its short-term inpatient use. Tofacitinib has less supporting evidence, but the STOP-COVID trial revealed a significantly reduced risk of death or respiratory failure over a 28-day period (Guimarães et al., 2021). While the involvement of the IL-23/IL-17 axis is evident in severe COVID-19, no established clinical trial data support using IL-17 or IL-23 antibodies for treatment at this time; only a few early-stage initiatives exist (Bonifácio et al., 2023). Beyond antiviral and immunomodulatory strategies used in hospitalized COVID-19 patients, growing attention has focused on how targeted biologic therapies for immune-mediated inflammatory diseases influence COVID-19 susceptibility and clinical outcomes.

Notably, patients with psoriasis who received anti-IL-12/23 antibodies, such as ustekinumab, completely recovered from COVID-19 without requiring hospitalization (Brownstone et al., 2020). While further research is needed, as the study included only two patients, it is reassuring that patients receiving biologic therapy for psoriasis can recover from COVID-19 infection. In contrast, an increased risk of COVID-19 was found in rheumatoid arthritis patients treated with anti-TNF-α therapy (Bagheri-Hosseinabadi et al., 2023). Furthermore, a recent study from Yu-Xin Zhen et al. demonstrated that patients treated with the IL-17 antibody ixekizumab were less likely to experience psoriasis exacerbation following COVID-19 infection (Zheng et al., 2024). However, another study found no significant differences in IL-17 levels between COVID-19 and healthy subjects (Orlov et al., 1950). Taken together, these findings highlight the complex and context-dependent effects of cytokine-targeted therapies in COVID-19, underscoring the need for larger, controlled studies to clarify their safety and potential therapeutic or protective roles across different patient populations.

## 3 Discussion

IL-23 has emerged as an important immunoregulatory cytokine linking inflammation, immune dysregulation, and tissue remodeling across diverse pulmonary diseases, including asthma, chronic obstructive pulmonary disease, idiopathic pulmonary fibrosis, and COVID-19. Although these disorders differ in etiology and pathology, they share convergent mechanisms involving IL-23–driven activation of Th17 and Th2 pathways, innate lymphoid cells, and downstream cytokines that perpetuate inflammation, airway remodeling, and fibrotic progression.

In asthma, IL-23 functions as a key upstream mediator bridging T2-high and non-T2 endotypes. It enhances Th17 and Th2 activity, leading to eosinophilic and neutrophilic inflammation, mucus hypersecretion, and corticosteroid resistance through JAK2–TYK2–STAT3 signaling (Agache and Akdis, 2016; Peng et al., 2010). Elevated IL-23 levels correlate with severe, mixed-granulocytic asthma and reduced corticosteroid responsiveness, underscoring its importance as both a biomarker and therapeutic target (Hastie et al., 2018; Ricciardolo et al., 2017; Wu and Peebles, 2023). Similarly, in COPD, cigarette smoke and microbial stimuli activate antigen-presenting cells to produce IL-23, which promotes Th17 polarization and ILC3 activation (Vassallo et al., 2010; Rouzic et al., 2017). This cascade sustains neutrophil recruitment via epithelial chemokines (CXCL1, IL-8) and contributes to airway destruction and emphysematous remodeling (Tian et al., 2024). Increased IL-23 levels in sputum and serum correlate with airflow limitation, GOLD stage, and corticosteroid resistance (Rong et al., 2020; Di Stefano et al., 2009; Liang et al., 2024), linking IL-23 to disease progression and inflammatory persistence.

In IPF, traditionally viewed as a fibrotic rather than inflammatory disorder, IL-23 has been implicated in fibroblast activation and epithelial–mesenchymal transition. Experimental studies show that IL-1β–induced IL-23 and IL-17A promote neutrophil infiltration, extracellular matrix accumulation, and fibrosis (Gasse et al., 2011). IL-23 also enhances the mesenchymal phenotype of alveolar epithelial cells, increasing collagen synthesis, αSMA expression, and resistance to apoptosis (Gasse et al., 2011; Senoo et al., 2021; Zhang et al., 2021). Pharmacological inhibition of IL-23 or its downstream IL-17 axis significantly attenuates fibrosis in preclinical models, suggesting that IL-23 contributes to both inflammatory and fibrogenic components of IPF pathogenesis (Gasse et al., 2011; Senoo et al., 2021; Zhang et al., 2021; Yin et al., 2020; Liu et al., 2023; Gu et al., 2023; Lv et al., 2025; Bellamri et al., 2023). However, in IPF, IL-23 may act predominantly as a disease-modifying amplifier of fibrogenic signaling rather than as a primary initiating driver of fibrosis.

During the COVID-19 pandemic, IL-23 has been identified as a contributor to the cytokine storm associated with severe disease. Elevated IL-23 levels correlate with disease severity and are accompanied by increased IL-17, IFN-γ, and Th17 activity (Smail et al., 2023; Markovic et al., 2021; Jovanovic et al., 2023), implicating this axis in hyperinflammatory lung injury. Genetic studies have also linked TYK2 expression, a downstream mediator of IL-23 signaling, with life-threatening COVID-19 outcomes (Pairo-Castineira et al., 2021). While direct IL-23 blockade remains unexplored clinically, JAK inhibitors such as baricitinib, which target IL-23 downstream signaling, have demonstrated improved survival and recovery in severe COVID-19 (Marconi et al., 2021; Recovery Collaborative Group, 2022; Kalil et al., 2021), reinforcing the therapeutic relevance of modulating this pathway. Nevertheless, in COVID-19, IL-23 likely functions as part of a broader inflammatory network and may amplify disease severity rather than act as a single dominant pathogenic driver.

Collectively, these findings position IL-23 as a mechanistically informative immunopathogenic axis across mechanistically distinct chronic and acute lung diseases, providing a conceptual framework that links inflammatory, corticosteroid-resistant, and fibrotic endotypes. By integrating evidence across asthma, COPD, IPF, and COVID-19, this review highlights shared and disease-specific IL-23–driven molecular signatures that can be leveraged for endotype-based patient stratification. Targeting IL-23 and its downstream pathways therefore represents a biologically rational, endotype-specific strategy for mechanism-based intervention in biologically defined patient subgroups with refractory respiratory disease. Moreover, these insights support the incorporation of IL-23–related biomarkers into molecular endotyping frameworks to guide treatment selection and future trial design in heterogeneous inflammatory and fibrotic lung disorders. Importantly, the strength of evidence supporting a causal role for IL-23 differs across disease entities and is currently strongest in asthma and COPD, whereas in IPF and COVID-19, IL-23 may act as a disease-modifying amplifier rather than a primary driver of pathology.

## 4 Limitations and future perspectives

Although IL-23 antibodies are effective in treating psoriasis and autoimmune conditions, pharmacovigilance data indicate that infections and paradoxical inflammatory reactions such as new or worsening skin conditions are common adverse effects, highlighting the immunosuppressive risks involved (Shi et al., 2025; Loft et al., 2020). Other safety reviews have raised concerns regarding less well-characterized long-term risks, such as potential cardiovascular safety signals observed in some datasets. This underscores the need for extended follow-up to determine the systemic effects of long-term IL-23/IL-12/23 pathway inhibition (de Brito and Yiu, 2021). Importantly, safety and efficacy profiles derived largely from dermatologic indications may not directly translate to chronic lung disease, where barrier immunity, microbial exposure, and infection susceptibility differ substantially. Although IL-23 inhibition demonstrates therapeutic potential, it also raises important safety and mechanistic concerns. Because IL-23 sustains Th17 cells and IL-17 signaling, long-term inhibition may weaken mucosal defense and increase the risk of bacterial and fungal infections, particularly in COPD and severe asthma patients already prone to airway infections and exacerbations (Wu and Peebles, 2023). Although CS- and elastase-induced emphysema models in mice demonstrated that IL-23-neutralizing antibodies can reduce disease severity (Tian et al., 2024), there is currently no clinical evidence supporting IL-23 antibodies in COPD, and they are limited by the risks of increased infections resulting from reduced Th17-mediated antimicrobial defense (Tan and Rosenthal, 2013). Furthermore, inhibiting IL-23 alone might not be enough in diseases caused by multiple cytokine networks (such as TNF-α and IL-6), leading to incomplete responses in certain patient groups (Krueger et al., 2024). Prolonged suppression might also lead to changes in immune responses, which could weaken treatment effectiveness or cause unexpected inflammatory reactions (Liu et al., 2020). In conditions dominated by fibrosis, like IPF, targeting IL-23 might provide limited benefits because the established fibrosis is usually sustained by structural remodeling rather than ongoing immune inflammation (Sisto and Lisi, 2023). Similarly, in severe COVID-19, IL-23 blockade might reduce cytokine-driven lung injury but disruption of antiviral immunity could be a major drawback, given the limited clinical evidence available.

In addition, monoclonal antibodies and emerging small-molecule inhibitors targeting the IL-23 pathway may entail distinct pharmacologic tradeoffs, including differences in target specificity, off-target effects, tissue penetration, and duration of immunosuppression, which could translate into divergent safety profiles and clinical risk–benefit balances across disease contexts. Because IL-23 pathway dependence likely varies across inflammatory endotypes, indiscriminate pathway inhibition may expose non–IL-23-driven patients to immunosuppressive risk without corresponding therapeutic benefit, underscoring the importance of biomarker-guided patient stratification and long-term pharmacovigilance. Collectively, these considerations suggest that IL-23–targeted therapies may have a context-dependent and potentially narrow therapeutic window in chronic lung disease, reinforcing the need for long-term safety monitoring and careful patient selection.

Building on these limitations, since its discovery, IL-23 has emerged as a key regulatory cytokine in immune-mediated inflammatory diseases. Its pathogenic role has been well established in autoimmune disorders, where IL-23 inhibitors are already in clinical use for conditions like psoriasis and IBD (Inflammatory bowel disease). More recently, both clinical and preclinical studies have demonstrated a crucial role for IL-23 and the IL-23/IL-17 axis in respiratory inflammatory diseases. Despite this progress, several limitations remain in fully elucidating the therapeutic potential of IL-23 targeting in pulmonary diseases. Future research should prioritize precise patient stratification to identify IL-23–driven inflammatory and fibrotic endotypes, enabling precision-guided, stratified therapeutic strategies rather than a one-size-fits-all strategy. Integrating multi-omics technologies—such as transcriptomics, proteomics, metabolomics, and single-cell sequencing—will be essential to unravel the heterogeneity of IL-23 signaling across disease types and progression stages. Longitudinal studies are also needed to determine whether IL-23 acts as an initiating driver of disease pathogenesis or as a secondary amplifier of chronic inflammation. Further, elucidating the interplay between IL-23 and other cytokine pathways, including IL-12, IL-1β, IL-6, and TGF-β, will be critical to understanding its context-dependent functions in the lung microenvironment. The influence of environmental exposures, comorbidities, and the lung microbiome on IL-23 expression and activity also remains largely unexplored and could reveal novel regulatory mechanisms of airway and parenchymal inflammation. Finally, the development of robust biomarkers that reflect IL-23 pathway activation would greatly enhance patient selection and monitoring of therapeutic efficacy in clinical trials. Such biomarkers, coupled with molecular endotyping, could enable precision-guided strategies that tailor IL-23–targeted interventions to specific inflammatory or fibrotic profiles.

### 4.1 Development of IL-23–related biomarkers and precision-guided treatment strategies

Based on our current understanding, non-invasive strategies for IL-23 biomarker development in respiratory diseases should focus on measuring IL-23 and Th17 mediators (e.g., IL-17, IL-22) using blood, sputum, and other airway-accessible samples, through methods like ELISA or cytokine panels, in combination with transcriptomic and proteomic profiling, rather than relying solely on lung biopsies. In COPD, serum IL-23 correlates with disease severity (Rong et al., 2020). Similarly, serum IL-23 levels are elevated in IPF, especially in IPF patients with lung cancer (Zhang et al., 2022). In asthma, serum IL-23 levels have been associated with disease severity in pediatric cohorts (Ciprandi et al., 2012b), and experimental models indicate that IL-23 contributes to airway inflammation and may reflect distinct inflammatory endotypes, supporting its inclusion in biomarker panels for immune phenotyping (Park and Lee, 2024). Together, these findings support the inclusion of IL-23 and Th17 mediators in cytokine panels to enable prognostic and patient-stratification utility in selected clinical subgroups.

Integrating IL-23-related biomarkers into molecular typing (endotyping) frameworks can facilitate more precise treatment by classifying patients into distinct inflammatory subgroups. Patients with an IL-23-high/Th17-dominant immune profile marked by elevated IL-23, IL-17, IL-22, and neutrophilic airway inflammation are likely to exhibit a higher inflammatory burden and reduced responsiveness to corticosteroids. These patients could be rationally selected candidates for IL-23/IL-17 axis–targeted interventions in biomarker-enriched clinical trials. Conversely, IL-23-low but type-2 dominant (eosinophilic/Th2-driven) patients are more likely to respond to established biologics targeting IL-4, IL-5, or IgE, rather than IL-23 blockade alone. Accordingly, IL-23 biomarker profiling may support diagnosis and prognosis and guide treatment selection by predicting therapeutic responsiveness in diverse inflammatory and fibrotic lung diseases (Sisto and Lisi, 2023). Importantly, IL-23 pathway activation should be interpreted in the context of broader immune networks, and biomarker panels should be used to identify IL-23–dependent disease biology rather than assuming uniform pathway relevance across patients.
